# Addressing Ageism—Be Active in Aging: Study Protocol

**DOI:** 10.3390/jpm12030354

**Published:** 2022-02-25

**Authors:** Júlio Belo Fernandes, Catarina Ramos, Josefa Domingos, Cidália Castro, Aida Simões, Catarina Bernardes, Jorge Fonseca, Luís Proença, Miguel Grunho, Paula Moleirinho-Alves, Sérgio Simões, Diogo Sousa-Catita, Diana Alves Vareta, Catarina Godinho

**Affiliations:** 1 Escola Superior de Saúde Egas Moniz, 2829-511 Almada, Portugal; cramos@egasmoniz.edu.pt (C.R.); ccastro@egasmoniz.edu.pt (C.C.); aidasimoes@gmail.com (A.S.); cbernardes@egasmoniz.edu.pt (C.B.); jorgedafonseca@hotmail.com (J.F.); sergio_23@netcabo.pt (S.S.); cgcgodinho@gmail.com (C.G.); 2 Grupo de Patologia Médica, Nutrição e Exercício Clínico (PaMNEC), Centro de Investigação Interdisciplinar Egas Moniz (CiiEM), 2829-511 Almada, Portugal; domingosjosefa@gmail.com (J.D.); miguelgrunho@gmail.com (M.G.); pmoleirinhoa@gmail.com (P.M.-A.); diogo.rsc2@gmail.com (D.S.-C.); 3 Instituto Universitário Egas Moniz, 2829-511 Almada, Portugal; lproenca@egasmoniz.edu.pt; 4 LabPSI, Centro de Investigação Interdisciplinar Egas Moniz (CiiEM), 2829-511 Almada, Portugal; 5 Centro de Investigação Interdisciplinar Egas Moniz (CiiEM), 2829-511 Almada, Portugal; 6 Department of Gastroenterology, Hospital Garcia de Orta EPE (HGO), 2805-267 Almada, Portugal; 7 Department of Neurology, Hospital Garcia de Orta, 2805-267 Almada, Portugal; 8 Sleep and Temporomandibular Disorder Department, Cuf Tejo Hospital, 1350-352 Lisboa, Portugal; 9 Department of Nursing, Centro Hospitalar de Setúbal, 2900-182 Setúbal, Portugal; diana_vareta@hotmail.com

**Keywords:** ageism, stereotyping, prejudice, discrimination, older adults, assessment, aging

## Abstract

Ageism refers to stereotyping (how we think), prejudice (how we feel), and discrimination (how we act) against people based on their age. It is a serious public health issue that can negatively impact older people’s health and quality of life. The present protocol has several goals: (1) adapt the Ambivalent Ageism Scale for the general Portuguese population and healthcare professionals; (2) assess the factorial invariance of the questionnaire between general population vs. healthcare professionals; (3) evaluate the level of ageism and its predictors in the general population and evaluate the level of ageism and its predictors in healthcare professionals; (4) compare the levels of ageism between groups and the invariance between groups regarding the explanatory model of predictors of ageism. This quantitative, cross-sectional, descriptive, observational study will be developed in partnership with several Healthcare Professional Boards/Associations, National Geriatrics and Gerontology Associations, and the Universities of the Third Age Network Association. The web-based survey will be conducted on a convenience sample recruited via various social media and institutional channels. The survey consists of three questionnaires: (1) Demographic data; (2) Ambivalent Ageism Scale; (3) Palmore-Neri and Cachioni questionnaire. The methodology of this study will include translation, pilot testing, semantic adjustment, exploratory and confirmatory factor analysis, and multigroup analysis of the Ambivalent Ageism Scale. Data will be treated using International Business Machines Corporation (IBM^®^) Statistical Package for the Social Sciences (SPSS) software and Analysis of Moment Structures (AMOS). Descriptive analysis will be conducted to assess the level of ageism in the study sample. The ageism levels between the two groups will be compared using the t-student test, and two Structural Equation Modeling will be developed to evaluate the predictors of ageism. Assessing ageism is necessary to allow healthcare professionals and policymakers to design and implement strategies to solve or reduce this issue. Findings from this study will generate knowledge relevant to healthcare and medical courses along with anti-ageism education for the Portuguese population.

## 1. Introduction

The demographic aging of the population is one of humanity’s greatest triumphs and challenges. By 2050 the number of people aged 60 or older is expected to rise from 962 million in 2017 to 2.1 billion in 2050 and 3.1 billion in 2100 [1].

In the European Union, Portugal has the fourth-highest percentage age of older people [2]. In 2000, the Portuguese population aging index was 98.8%, having almost doubled in 2018, to 157.7%, increasing the dependency index from 24% to 33.6% [3].

Aging becomes a problem when society is not prepared for its own aging, presenting negative attitudes towards this stage of life [4].

Ageism, a term first coined by Robert Butler, refers to stereotyping (thoughts), prejudice (feelings), and discrimination (actions or behavior) against people by their age [5], has been identified as a serious threat to active aging and a major public health issue [6]. Despite being described in the bibliography since the late 60s, it took more than 30 years for ageism to be addressed within human rights instruments like the Madrid International Plan of Action on Ageing [7]. It is a multidimensional concept that includes different dimensions (cognitive, affective, and behavioral) operating at a micro-level (singular person), meso-level (social networks), and macro-level (cultural or institutional) and can occur consciously (explicitly) or unconsciously (implicitly) [4].

Ageism can have different targets, as it can be directed towards others or oneself [8]. In addition, ageism can also affect people of different ages, but children, adolescents, and older adults feel it more frequently. In our study, we intend to obtain data about the age-based attitudes and discrimination affecting older adults, and therefore the term “ageism” used here is applied for this context.

Definitions of ageism refer to older people, aging, and the aging process as being seen in an undesirable fashion. Ageist attitudes are of great complexity as they fit the paternalistic stereotype. Older adults are seen as warm but incompetent, ending in hostile and benevolent forms of prejudice [9,10]. Because most people consider the manifestation of protective behaviors and attitudes towards older adults positive, it is more challenging to address the benevolent forms of ageism [9,10].

Numerous studies have verified that ageism negatively impacts older people in several distinct dimensions such as memory and cognitive performance [11,12], health and wellbeing [13], social isolation and loneliness [14], job performance [15], decreased quality of life [13], and even their will-to-live [16]. These findings suggest that negative stereotypes, prejudice, or discrimination against people by their age can become internalized to such an extent that conscious or unconscious influence the person’s cognitive and/or physical capacity.

While negative representations of older adults may lead to exclusion, hostile attitudes and behaviors [8,9], and social exclusion [17], favorable depictions often provoke well-intended benevolent behaviors, like providing unneeded support [18,19].

It is common to find ageism in many sectors of society, including those providing health and social care. Considering healthcare systems, older adults represent a significant group of users, and their care has a crucial impact on the overall financial costs [20]. However, these systems are designed considering the care needs of a younger population, aiming to achieve a quick turnover and not prioritizing the complexity of older adults’ health and social concerns. Several studies involving healthcare professionals support that ageism contributes to worse received care for older adults with poorer health outcomes [21,22]. Conversely, healthcare professionals who have positive perceptions of older adults are more prone to assess and manage their healthcare concerns and social needs [23]. Additionally, if we consider that health professionals broadly foster a paradigm shift towards active aging, it becomes essential to assess the presence of ageism in this population as ageism may be reflected in their clinical practice. Health professionals must take the lead in promoting active aging and ensuring that their care practices enable and empower older adults to remain as autonomous and independent as possible for as long as possible [24]. Therefore, it is crucial to assess the presence of ageism and its predictors in the population, with a special focus on healthcare professionals.

The research will have two phases. Phase 1 aims to evaluate ageism in Portuguese healthcare professionals and identify its predictors. In Phase 2, we will expand the study to a representative sample of the Portuguese population.

We chose the Ambivalent Ageism Scale to evaluate ageism because it is a useful tool for researchers to measure attitudes toward older adults, allowing us to assess different elements of ageist attitudes and benevolent and hostile ageism [9].

### 1.1. Research Questions

(a) What is the level of ageism in Portuguese healthcare professionals?
(b) What are the predictors of ageism in Portuguese healthcare professionals?
(c) What is the level of ageism in the Portuguese population?
(d) What are the predictors of ageism in the Portuguese population?

### 1.2. Objective

Given the research questions mentioned above, the study goals are: (a) adapt the Ambivalent Ageism Scale for the general Portuguese population and for healthcare professionals; (b) assess the factorial invariance of the questionnaire between both groups (general population vs. healthcare professionals); (c) evaluate the level of ageism and its predictors in the general population; (d) evaluate the level of ageism and its predictors in healthcare professionals; (e) compare the levels of ageism between groups and the invariance between groups regarding the explanatory model of predictors of ageism.

## 2. Methods

### 2.1. Design

A quantitative, cross-sectional, descriptive, observational study conducted based on a web-based survey.

### 2.2. Time Period

**Figure 1:**
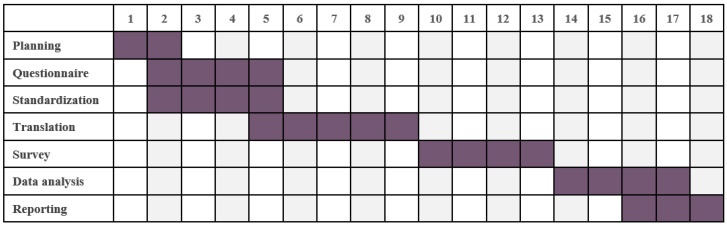
Project schedule.

January 2022–June 2024 (Gantt Chart Figure 1).

### 2.3. Population and Recruitment

We will use a convenience sampling to recruit participants via various social media and institutional channels.

#### 2.3.1. Phase 1

The study population consists of healthcare professionals (i.e., nurses, physiologists, physiotherapists, psychologists, speech therapists, occupational therapists, and doctors).

#### 2.3.2. Phase 2

The study population consists of Portuguese citizens.

### 2.4. Inclusion Criteria

#### 2.4.1. Phase 1

- Portuguese health care professional’s (i.e., Portuguese nurses, physiologists, physiotherapists, psychologists, speech therapists, occupational therapists, doctors);
- Willingness to participate in the study.

#### 2.4.2. Phase 2

- Portuguese population;
- People aged 18 years and above;
- Willingness to participate;
- Ability to understand, provide informed consent and comply with all the proceedings.

### 2.5. Exclusion Criteria

Target population under the age of 18, and an unwillingness/inability to understand, provide informed consent, or comply with all the proceedings.

### 2.6. Sample Size Calculation

The total sample consists of 168 participants, which will be divided into 2 groups (84 healthcare professionals and 84 participants from general population from Portugal). The sample size was obtained based on a power calculation (5% level of significance) indicating that a total sample size of 140 will have 95% power, for a small effect size (0.25). Therefore, sample size was increased to 168 participants to accommodate a 20% of exclusions.

### 2.7. Partner Institutions

In Phase 1, the study will be conducted in collaboration with the Board of Nurses, Board of Physiotherapists, Board of Psychologists, Board of Medicine, and the Portuguese Association of Exercise Physiologists.

These partners will use their database to forward an email (with a link to access the survey), inviting their members/associates to participate in the study. At the same time, we will create a campaign to publicize the study on different social media channels to maximize the number of respondents.

In phase 2 the study will be conducted in collaboration with the National Association of Social Gerontology, the Portuguese Society of Geriatrics and Gerontology, and the Universities of the Third Age Network Association. In addition, we will start a campaign to publicize the study on different social media channels to maximize the number of respondents.

### 2.8. Data Collection

Data collection will be carried out through the application of the web-based survey using Qualtrics. Participants will answer the survey. At the end of all the questionnaires, participants can see the contact details of the researchers for any questions or comments, as well as links to other organizations that provide information or intervention in the area of ageism. The survey’s first page will clarify the objectives and procedures of the study and the guarantee to ensure the confidentiality and anonymity of the data. Before completing the survey, informed consent will be obtained from all study participants.

### 2.9. The Survey

Part 1—Demographic data: Multiple choice and quick answer questions are presented to allow the sociodemographic characterization of the sample. Participants will report information such as gender, age, nationality, education, professional status, frequency, and quality of intergenerational contact.

Part 2—Ambivalent Ageism Scale: To assess the presence of benevolent and hostile ageism, we will use the Ambivalent Ageism Scale developed by Cary, Chasteen, and Remedios [9]. Participants will report their agreement on a 7-point Likert-type scale with 13 statements regarding older adults. Nine of those items allow assessing the presence of benevolent ageism, and the remaining four items the presence of hostile ageism (e.g., “It is helpful to repeat things to old people because they rarely understand the first time.”, “It is good to tell old people that they are too old to do certain things; otherwise they might get their feelings hurt when they eventually fail.” or “Old people are a drain on the health care system and the economy.”).

Part 3—Palmore-Neri and Cachioni questionnaire: To assess the level of knowledge concerning aging, we will use the Palmore-Neri and Cachioni questionnaire [25]. The questionnaire is an adaptation of the Palmore Aging Quiz, consisting of 25 items covering general knowledge about aging and questions about older adults’ physical, psychological, and social dimensions.

Ageism and level of knowledge are constructs that can be influenced by social desirability. Therefore, to control for social desirability bias, we will include a short text before the presentation of the questionnaires (based on the text from Goetzke, Nitzko, and Spiller [26]). In addition, we will also apply the Balanced Inventory of Desirable Responding (BIDR) short scale (Winkler, Kroh, & Spiess [27]; adapted to English by Goetzke, Nitzko, and Spiller [26]), as a complementary measure to control for social desirability bias.

### 2.10. Ambivalent Ageism Scale Translation and Cultural Validation

The Ambivalent Ageism Scale will be translated in accordance with World Health Organization best practice guidelines [28].

The translation process will be performed through different stages, namely: forward translation, an expert panel, back-translation, pre-testing, and cognitive interviewing. Our team has already gained permission by email correspondence to use and translate the Ambivalent Ageism Scale. Translation and cultural validation will take place between May 2022 and September 2022.

#### 2.10.1. Stage I—Forward Translation

The translation of the questionnaire from the original English version into the Portuguese language will be accomplished by two independent bilingual translators. One translator will be an expert on Ageism, providing a translation that more closely resembles the original instrument. The other will be a naïve translator, who is unaware of the questionnaire’s aim, producing another translation so that subtle variances in the original questionnaire might be perceived. Any divergences in the translation will be debated and resolved between the two translators or, if needed, a third unbiased translator fluent in both languages. With the forward translation, we aim to translate the questionnaire and determine its conceptual equivalence in Portuguese, reflecting the nuances of the target language.

#### 2.10.2. Stage II—Back-Translation

The back-translation will be performed independently from the forward translation to ensure the accuracy of the translation. This stage will be done independently by two further translators fluent in both Portuguese and English. Following the best practice recommendation, these two translators will not have previous exposure to the original questionnaire. As in the previous stage, any divergences in the translation will be debated and resolved between the two translators or, if needed, a third unbiased translator. In this stage, we aim to reveal any misunderstandings or unclear wording in the initial translation.

#### 2.10.3. Stage III—Questionnaire Pilot Test and Semantic Adjustment

The translated version will be applied to a small (34, 20%, from the total sample), randomly selected sample of men and women to discuss the items’ understanding and arrive at the final translation of the Portuguese version of the questionnaires Ambivalent Ageism Scale. The Ambivalent Ageism Scale will be pilot tested through a think-aloud protocol [29] as per the best practice recommendation. Participants will be asked to fill out the questionnaire while verbalizing their thought processes. This process enables researchers to gather information on how participants understood the questionnaire and its instructions and allows the verification of the questionnaire comprehensibility for Portuguese speakers.

Subsequently, researchers will apply the instruments to the general and healthcare samples within the scope of the investigation protocol.

### 2.11. Data Analysis

Data will be treated using Statistical Package for the Social Sciences (SPSS) software (International Business Machines Corporation (IBM) SPSS Statistics^®^, v.27.0, IBM^®^ Corp, Armonk, NY, USA) and Analysis of Moment Structures (AMOS^®^) (v.27.0, SPSS Inc., Chicago, IL, USA). To assess the construct validity of the Ambivalent Ageism Scale, exploratory factor analyses (EFA) will be performed. The principal components method will be used for the extraction of common factors and the Varimax rotation for the factor rotation [30]. Confirmatory factor analyses will be performed using Structural Equation Modeling (SEM) with Maximum Likelihood as estimator. Confirmatory factor analyses models will be evaluated with chi-square, and an alpha level of 0.05 will be used to determine statistical significance. Model fit will be assessed using the comparative fit index (CFI), the goodness of fit index (GFI), and the root mean square error of approximation (RMSEA). Values above 0.90 on the CFI and the GFI and below 0.05 on the root mean square error of approximation are indicators of good model fit [31]. Model invariance between groups (general population and healthcare professionals samples) regarding baseline model and resulting models will be calculated using ΔCFI (≤ 0.01) [31]. After the final version of the Ambivalent Ageism Scale is obtained, a descriptive analysis (i.e., mean, standard deviation, minimum, maximum) of ageism will be conducted to assess the level of ageism in both groups. The ageism levels between the two groups will be compared using the t-student test. In order to evaluate the predictors of ageism, two versions of Structural Equation Modeling will be developed, one for the general population sample and the other for the healthcare professionals sample, with ageism as the dependent variable and with the following variables as independent variables: level of knowledge concerning aging, gender, age, nationality, education, professional status, frequency, and quality of intergenerational contact. The same criteria mentioned above will be used to evaluate the model fit. The invariance between the Structural Equation Modeling models of ageism predictors of the general population and the healthcare professionals will be assessed through a multigroup analysis. The same criteria for assessing model invariance mentioned above will be applied.

### 2.12. Ethics and Procedures

This research will be conducted in accordance with the Helsinki Declaration (as revised in 2013) and will seek approval from the Egas Moniz Ethics Committee.

All the participants must complete an informed consent question embedded on the first page of the questionnaire. It will state that participation is entirely voluntary. Participants are also free to not reply to some questions, change or review their responses, or voluntarily quit at any time. Consent will be obtained before proceeding to the next page if participants answer “YES” to the form’s first question, agreeing to participate in the study. Participants who answer “NO” to the informed consent question will be directed to the end of the survey. Data will be conducted in compliance with ethical principles guaranteeing the participants’ anonymity. Therefore, no individual answers to the questionnaire will be accessible.

### 2.13. Confidentiality and Data Retention

In this study, all data collected from participants will be strictly anonymous and confidential. Researchers are not interested in individual responses. Only the project managers will have access to all data. Essential documents will be archived in a way that ensures that they are readily available, upon request, to the competent authorities. All paper copies will be stored in a locked file. Data collected from the survey will be coded and stored on a password-protected and backed-up computer drive. For five years, all data will remain locked in a file cabinet at Egas Moniz University. The project managers will destroy all data when this retention period is complete.

## 3. Discussion

This study represents a starting point to change the narrative around age and aging among the Portuguese population. Up to now, there has been no significant study that addresses ageism in Portugal. Ageism can harm members of society individually and collectively, with negative consequences for their health and wellbeing, being responsible for a heavy economic burden on society. For example, in the United States of America, age stereotypes led to excess annual costs of US$63 billion for the eight most expensive health conditions [32]. Ageist behaviors can be combatted. However, collective action is needed to increase awareness and address this issue for this to happen. Increasing peoples’ awareness of ageist behaviors is essential to decreasing ageism as a persistent social phenomenon.

There are several scales designed to measure hostile ageism, potentially making it difficult for clinicians to decide which to choose for better practices. Notably, the Ambivalent Ageism Scale is the first dedicated to measuring both hostile and benevolent ageism since hostile and benevolent ageist attitudes exist and do not predict the same outcomes [9]. This scale had good test-retest reliability (r = 0.80) and good internal consistency (α = 0.91). The internal consistency of the benevolent subscale was α = 0.89, for the hostile ageism subscale α = 0.84 [9]. Besides being a useful tool for researchers to assess hostile and benevolent ageism, respondents do not perceive their participation in the survey as difficult or time-consuming when compared to the Ambivalent Ageism Scale (consisting of report agreement with 13 statements) [9].

The data collected in this study may play a central role in raising awareness of ageism among the Portuguese population, with a particular focus on healthcare professionals. After its completion, it is expected that the team of researchers will work with key partners to develop content related to ageism and share this content on various social media platforms to raise awareness of this problem.

Assessing ageism is the first step that allows healthcare professionals and policymakers to develop and implement strategies (e.g., education strategies) to solve this issue. Previous studies investigated strategies to combat ageism. For example, research developed in Canada addressed the effect of educational programs on nursing students’ knowledge and ageist behaviors. Researchers concluded that education had a positive impact on the way students view older adults [33].

Other studies also show that increased aging knowledge was significantly correlated with reduced ageist behaviors [34,35,36]. Therefore, raising awareness about ageism followed by increasing knowledge about aging should be the strategy to minimize the burden of this public health problem. Furthermore, knowledge and skills can be transmitted by developing educational activities enhancing understanding and empathy regarding aging.

In addition, previous studies have shown that a high level of encounters in the right setting decreases ageism, so multidisciplinary intervention to promote intergenerational contact should be promoted [37,38,39]. There is a clear need to increase aging knowledge combined with open, natural, and mutual relationships that will enable the reduction of negative attitudes towards aging.

This study protocol presents our aims, methodological approach, and plan to operationalize the research. The findings are expected to have direct relevance to several healthcare and medical courses across Portugal, along with anti-ageism education for the general public.

It is necessary to develop the capacity to challenge, rather than perpetuate, stereotypes, prejudice, and discrimination against people by their age. Therefore, this study is of vital importance.

This study has the advantage of contributing to a more complete representation of ageism among the Portuguese population. Nevertheless, we emphasize that the study has limitations. The first is the possibility that participants might deny or minimize their ageist behaviors if they recognize them as incorrect or socially undesirable. Alternatively, if participants perceive it to be socially desirable, they might overstate the frequency of their behavior, increasing the frequency of the positive items. Second, by relying upon recruiting participants via various social media, we may not generalize the survey result to the population as a whole as there is the possibility of under-or over-representation.

## Data Availability

Not applicable.
